# Gene Polymorphisms of Novel Immunotolerant Molecule BTLA: Distribution of Alleles, Genotypes and Haplotypes in Polish Caucasian Population

**DOI:** 10.1007/s00005-014-0300-3

**Published:** 2014-09-03

**Authors:** Anna Partyka, Dariusz Woszczyk, Tomasz Strzała, Anna Szczepańska, Anna Tomkiewicz, Irena Frydecka, Lidia Karabon

**Affiliations:** 1 Department of Experimental Therapy, Institute of Immunology and Experimental Therapy, Polish Academy of Science, Weigla 12, 53-114 Wroclaw, Poland; 2 Department of Hematology, State Hospital, Opole, Poland; 3 Department of Genetics, Wroclaw University of Environmental and Life Sciences, Wroclaw, Poland; 4 Department of Clinical Urology, Wroclaw Medical University, Wroclaw, Poland

**Keywords:** Gene polymorphism, Co-inhibitory molecule, BTLA, Caucasian population

## Abstract

B and T lymphocyte attenuator (BTLA) is one of the members of immunoglobulin superfamily which, like CTLA-4 and PD-1, is involved in down regulation of immune response. Despite the important role of BTLA in maintaining immune homeostasis, relatively little studies were devoted to the relationship of polymorphisms in the gene encoding *BTLA* with susceptibility to autoimmune disease and cancer. Moreover, all published works were done in Asian populations. *BTLA* gene is located on chromosome 3 in q13.2 and consists of five exons. The aim of this study was to investigate the alleles, genotypes and haplotypes frequency of selected *BTLA* gene polymorphisms in Caucasian population originating from Poland. For this study, the single-nucleotide polymorphisms (SNPs) were chosen on the basis of literature data. Additionally, the tag dSNP under linkage equilibrium *r* ^2^ > 0.8 and available at the National Center for Biotechnology Information (NCBI) for Caucasian population of rare alleles at a frequency greater than 5 % have been chosen using the NCBI database. The ten *BTLA* SNPs investigated were: rs1844089, rs2705535, rs9288952, rs9288953, rs1982809, rs2633580, rs2705511, rs2705565, rs76844316, rs16859633. For all SNPs selected on the basis of literature data the significantly different distributions of genotypes between Asian and Caucasian populations were observed.

## Introduction

B and T lymphocyte attenuator (BTLA) is a suppressor molecule belonging to the immunoglobulin superfamily which, like cytotoxic leukocyte antigen-4 (CTLA-4) and programmed death-1 (PD-1), is involved in the inhibition of the immune response. BTLA shares only 9–13 % amino acid identity with CTLA-4 and PD-1, but is structurally similar to them and like other co-inhibitory molecules has two immunoreceptor tyrosine-based inhibitory motifs (ITIM) in its cytoplasmic region (Watanabe et al. 2003). BTLA interaction with the ligand herpes virus entry mediator (HVEM) causes phosphorylation of tyrosine within the ITIM and their connection to protein tyrosine phosphatase SHP-1 and SHP-2, and consequently inhibition of T-cell activation and production of cytokines interferon γ, interleukin (IL)-2, IL-4, and IL-10 (Murphy et al. 2006; Nelson et al. 2008; Sedy et al. 2005). BTLA-mediated inhibition of T-cell activation occurred during both the primary CD4^+^ and secondary CD4^+^ and CD8^+^ T-cell response, suggesting that BTLA ligation provides a constitutive inhibition signal to T cells (Otsuki et al. 2006). BTLA expression is equal on Th1 and Th2 cells (Otsuki et al. 2006).

BTLA expression in murine naive B cells is high, but its role in the activation and differentiation of B cells has not been studied. BTLA expression in naive murine T cells is negligible, but its expression increases during T-cell activation. BTLA expression is also significant in mice thymocytes during positive selection (Watanabe et al. 2003). Moreover, BTLA is expressed by macrophages, dendritic cells and natural killer cells (Han et al. 2004). In recent years strong expression of BTLA on malignant B cells in patients with B-cell chronic lymphocytic leukemia has been reported in the literature (M’Hidi et al. 2009).

Because BTLA was relatively recently described in the literature, there are only a few studies that address *BTLA* gene polymorphisms, and most have investigated its role in susceptibility to autoimmune diseases, such as rheumatoid arthritis (Lin et al. 2006; Oki et al. 2011), systemic lupus erythematosus and type 1 diabetes mellitus (Inuo et al. 2009) and only one in risk of cancer (Fu et al. 2010).

The aim of this study was to determine allele, genotype and haplotype frequencies as well as linkage disequilibrium of selected polymorphisms in the *BTLA* gene.

## Materials and Methods

DNA was isolated from venous blood from 417 unrelated healthy volunteers from Western Poland (172 female/245 male) according to the manual procedure for white blood cells using the QIAamp DNA Blood Mini Kit (Qiagen, Germany).

In Poland migration and nationality diversion is very small, therefore Polish cohort was homogenous. The genetic homogenization was reflected in virtually identical frequencies of H-Y polymorphisms in different regions of Poland described by Ploski et al. (2002).

### Genotyping

Nine single-nucleotide polymorphisms in the *BTLA* gene, rs1844089, rs2705535, rs9288952, rs9288953, rs1982809, rs2633580, rs2705511, rs76844316, rs16859633, were genotyped using the TaqMan^®^SNP Genotyping Assays, respectively: C__26921149_20, C__16272852_10, C__1175845_10, C__1175838_10, C__1175848_20, C__16047575_10, C__16272823_10, assay on demand, C__34010634_10, provided by Applied Biosystems (Foster City, USA). Genotyping for rs2705565 was done using TIB MOLBIOL LightSNiP assay (no. 24901301).

### Statistical Analysis

The evaluation of Hardy–Weinberg equilibrium (HWE) was performed by comparing the observed and expected frequencies of genotypes using *χ* ^2^ analysis. The haplotype frequencies for pairs of alleles were determined using the SHEsis program (http://202.120.7.14/analysis/myAnalysis.php) (Shi and He 2005). Haplotypes with frequencies lower than 0.03 were not considered. Linkage disequilibrium coefficients *r* ^2^ (values for a pair of the most common alleles at each locus) were estimated using http://202.120.7.14/analysis/myAnalysis.php (Shi and He 2005). The *χ* ^2^ test was used to compare categorical data between poles and other populations on the basis of literature data. Differences were considered statistically significant if *p* < 0.05. For the multiple comparisons, Bonferroni multiple adjustments were employed to the level of significance.

Furthermore, to analyze ethnic relationship between the Polish and other populations [Yoruba in Ibadan, Nigeria; Japanese in Tokyo, Japan; Han Chinese in Beijing, China; CEPH (Utah residents with ancestry from northern and western Europe)], we estimated phylogenetic tree based on nine out of ten single nucleotide polymorphism (SNP) *loci* (rs76844316 *locus* was excluded because of lack of data for non-Polish populations) used in present work. The tree was created using SNP allele frequency [for Polish population data were estimated in present study and for others possessed from Hapmap database (Genbank)] with Poptree 2 (Takezaki et al. 2010) using Neigbour Joining algorithm (Saitou and Nei 1987) and *D*_A_ distance (Nei et al. 1983) as a measure of population differentiation. The tree’s nodes significance was evaluated with 1,000 bootstrap analysis.

## Results and Discussion

We found that each polymorphism in the *BTLA* gene was in HWE.

Distributions of alleles and genotypes are presented in Table 1. In five out of ten investigated polymorphisms wild alleles were observed in more than 90 % of the Polish population. In the case of intron polymorphism rs9288953, we observed about 40 % minor allele frequency, while for rs1982809 situated in the 3′ near gene position and rs2705511 located in the intragenic region the minor allele frequency was about 24 % in both cases. Two other polymorphisms—rs76844316, described in the literature as polymorphic in the Japanese population (Oki et al. 2011); and rs16859633, chosen on the basis of HapMap analysis—seem not to be polymorphic in Poles since none of 200 genotyped volunteers were carriers of mutant alleles.

**Table 1:** Distribution of alleles and genotypes of the following SNPs: rs2705511, rs1982809, rs9288952, rs76844316, rs16859633, rs9288953, rs2705535, rs1844089, rs2705565, rs2633580 (listed in the physical position order; contig NT_005612.16)

SNP	Alternative name	Chromosome position	Location	Major allele	Minor allele	First homozygous *N* (%)	Heterozygous *N* (%)	Second homozygous *N* (%)
rs2705511	*g.18674625A* > *C*	112179479	Intragenic	**A** 622 (74.58)	**C** 212 (25.42)	234 (56.11)	154 (36.94)	29 (6.95)
rs1982809	*g.18677886A* > *G*	112182740	3′ near gene	**A** 636 (76.26)	**G** 198 (23.74)	245 (58.89)	146 (34.77)	26 (6.24)
rs9288952	*g.18680171G* > *A*	112185025	Exon 4 (Pro-Leu)	**A** 784 (94.00)	**G** 50 (6.00)	369 (88.50)	46 (11.02)	2 (0.48)
rs76844316	*g.18683755T* > *G*	112188609	Exon 4 Asn-Thr	**T** 400 (100)	**G** 0 (0)	200 (100)	0 (0)	0 (0)
rs16859633	*g.18693481T* > *C*	112198335	Exon 2 Ile-Val	**T** 400 (100)	**C** 0 (0)	200 (100)	0 (0)	0 (0)
rs9288953	*g.18698398C* > *T*	112203252	Intron 1	**C** 521 (62.47)	**T** 313 (37.53)	164 (39.32)	193 (46.28)	60 (14. 39)
rs2705535	*g.18704073C* > *T*	112208927	Intron 1	**C** 823 (98.68)	**T** 11 (1.32)	406 (97.36)	11 (2.64)	0 (0)
rs1844089	*g.18712880G* > *A*	112217734	Intron 1	**G** 758 (90.98)	**A** 76 (9.11)	343 (82.26)	72 (17.26)	2 (0.48)
rs2705565	*g.18714482C* > *T*	112219336	5′ near gene	**C** 736 (94.85)	**T** 40 (5.15)	349 (89.95)	38 (9.79)	1 (0.25)
rs2633580	*g.18714899C* > *G*	112219753	5′ near gene	**G** 759 (91.23)	**C** 73 (8.77)	346 (83.17)	67 (16.11)	3 (0.72)

In haplotype analysis two non-polymorphic sites, rs76844316 and rs16859633, were omitted. Haplotype frequencies are presented in Table 2. Only four haplotypes were represented in the Polish population with frequency >3 %. The haplotype of rs2705511A, rs1982809A, rs9288952A, rs9288953C, rs2705535C, rs1844089G, rs2705565C, rs2633580G, consisting of wild alleles appeared in almost 50 %.

**Table 2:** Analysis of estimated haplotype frequency of SNPs rs2705511, rs1982809, rs9288952, rs9288953, rs2705535, rs1844089, rs2705565, rs2633580 in Polish population

rs2705511	rs1982809	rs9288952	rs9288953	rs2705535	rs1844089	rs2705565	rs2633580	Haplotype frequency
A	A	A	C	C	G	C	G	0.476
A	A	A	T	C	G	C	G	0.209
C	G	A	T	C	G	C	G	0.122
C	A	A	C	C	G	C	G	0.035

The linkage equilibrium analysis showed that rs1844089 and rs2633580 as well as rs9288952 and rs2705565 were in strong linkage disequilibrium (*r* ^2^ = 0.90 and *r* ^2^ = 0.81, respectively). Moreover, the following pairs were in moderate linkage disequilibrium expressed by *r* ^2^ > 0.5: rs1844089 and rs9288952, rs9288952 and rs2633580, rs1982809 and rs2705511, rs1844089 and rs2705565, rs2633580 and rs2705565 (Table 3).

**Table 3:** Linkage disequilibrium coefficients *r* ^2^ values for pairs of the investigated SNPs

*r* ^2^	rs1982809	rs9288952	rs9288953	rs2705535	rs1844089	rs2705565	rs2633580
rs2705511	0.596	0.045	0.064	0.003	0.023	0.025	0.026
rs1982809	–	0.123	0.089	0.020	0.085	0.106	0.082
rs9288952	–	–	0.029	0.169	0.552	**0.810**	0.578
rs9288953	–	–	–	0.008	0.051	0.032	0.057
rs2705535	–	–	–	–	0.133	0.265	0.139
rs1844089	–	–	–	–	–	0.548	**0.900**
rs2705565	–	–	–	–	–	–	0.575

*r* ^2^ values with strong LD >0.8 are given in bold

Comparison with data available from HapMap (http://hapmap.ncbi.nlm.nih.gov/cgi-perl/gbrowse/hapmap24_B36/#search 2013) for different populations is presented in Table 4. Our results are similar to those reported in HapMap for a Caucasian population except for rs2705565. However, distribution of alleles in almost all investigated SNPs varies for Asian populations—Han Chinese and Japanese.

**Table 4:** Summary data on the frequency of alleles for BTLA gene polymorphisms in the Polish population from the present study and in other populations based on data from HapMap 2013 (http://hapmap.ncbi.nlm.nih.gov/cgi-perl/gbrowse/hapmap24_B36/#search)

SNP	Major allele	Minor allele	Poles present study		HapMap CEU		HapMap YRI		HapMap JPT		HapMap CHB	
			Major allele	Minor allele	Major allele	Minor allele	Major allele	Minor allele	Major allele	Minor allele	Major allele	Minor allele
rs2705511	A	C	0.746	0.254	0.670	0.330	0.650	0.350	0.216	0.784	0.267	0.733
rs1982809	A	G	0.763	0.237	0.733	0.267	0.683	0.317	0.170	0.830	0.233	0.767
rs9288952	A	G	0.940	0.060	0.974	0.026	0.110	0.890	0.716	0.284	0.733	0.267
rs76844316	T	G	1	0	–	–	–	–	–	–	–	–
rs16859633	T	C	1	–	1	0	0.983	0.017	1	0	1	0
rs9288953	C	T	0.625	0.375	0.658	0.342	0.992	0.008	0.420	0.580	0.456	0.544
rs2705535	C	T	0.987	0.013	0.982	0.018	0.839	0.161	0.822	0.178	0.798	0.202
rs1844089	G	A	0.910	0.090	–	–	0.342	0.658	0.739	0.261	0.756	0.244
rs2705565	C	T	0.949	0.051	0.942	0.058	–	–	0.818	0.182	0.825	0.175
rs2633580	G	C	0.912	0.088	0.940	0.060	0.347	0.653	0.739	0.261	0.756	0.244

*CEU* CEPH (Utah residents with ancestry from northern and western Europe), *YRI* Yoruba in Ibadan, Nigeria, *JPT* Japanese in Tokyo, Japan, *CHB* Han Chinese in Beijing, China

**Fig. 1:**
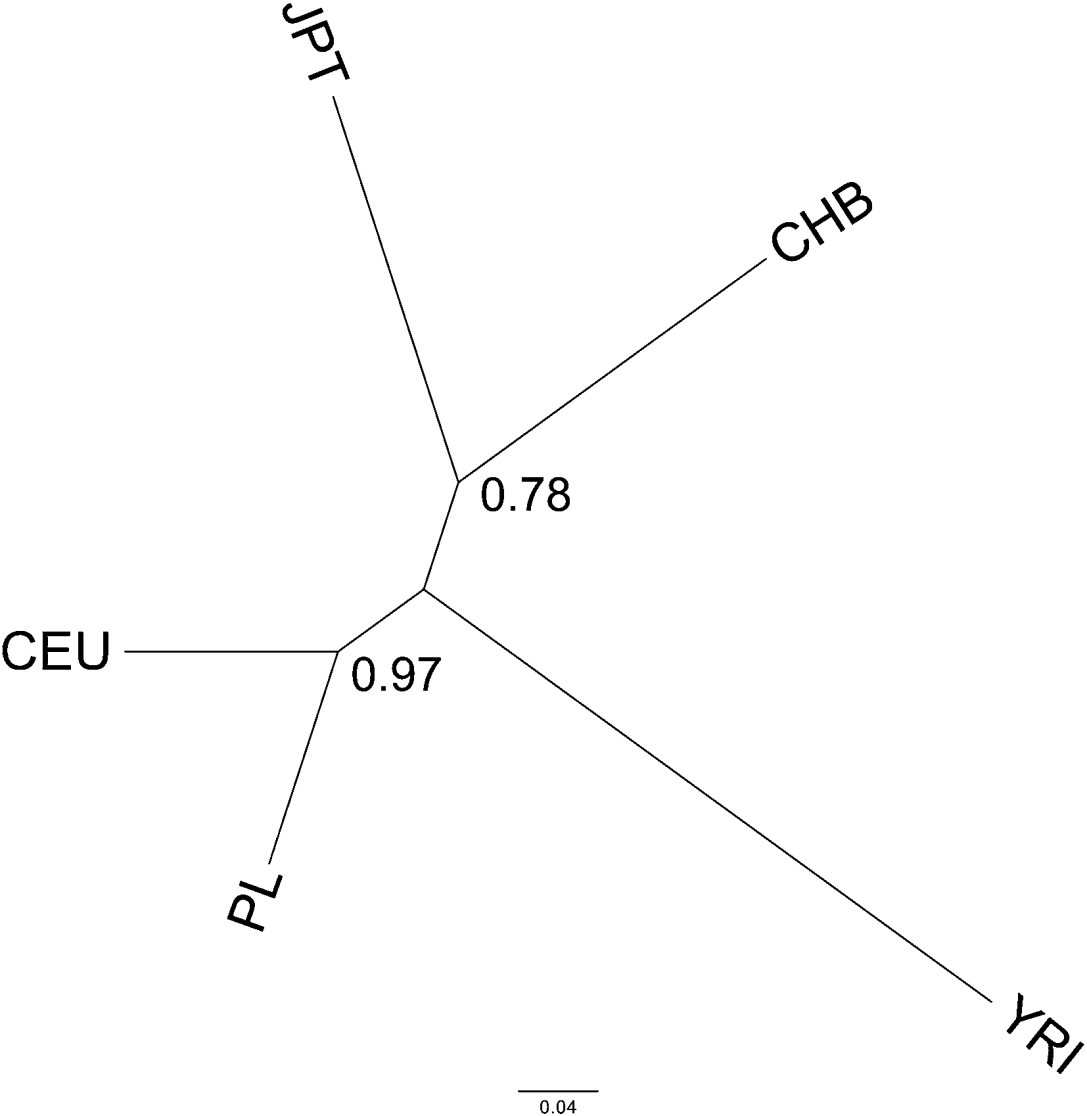
Phylogenetic tree presenting relationship between five ethnically different human populations (*CEU* CEPH (Utah residents with ancestry from northern and western Europe); *YRI* Yoruba in Ibadan, Nigeria; *JPT* Japanese in Tokyo, Japan; *CHB* Han Chinese in Beijing, China) created with SNP frequency data. Numbers along nodes are bootstrap values

Phylogenetic tree obtained in our study showed clear division into three clades (Fig. 1). First clade was consisted of Polish and Caucasian (CEU) populations, second with Japanese (JPT) and Chinese (CHB) populations and third one with African population (YRI). Obtained results indicated that Polish population is ethnically and genetically closest to Caucasian population (represented in this analysis by CEPH population which was derived from European ancestors). Furthermore, both Caucasian populations were different from Japanese and Chinese populations (which were closest to each other) and all aforementioned populations were distinct from African population.

**Fig. 2:**
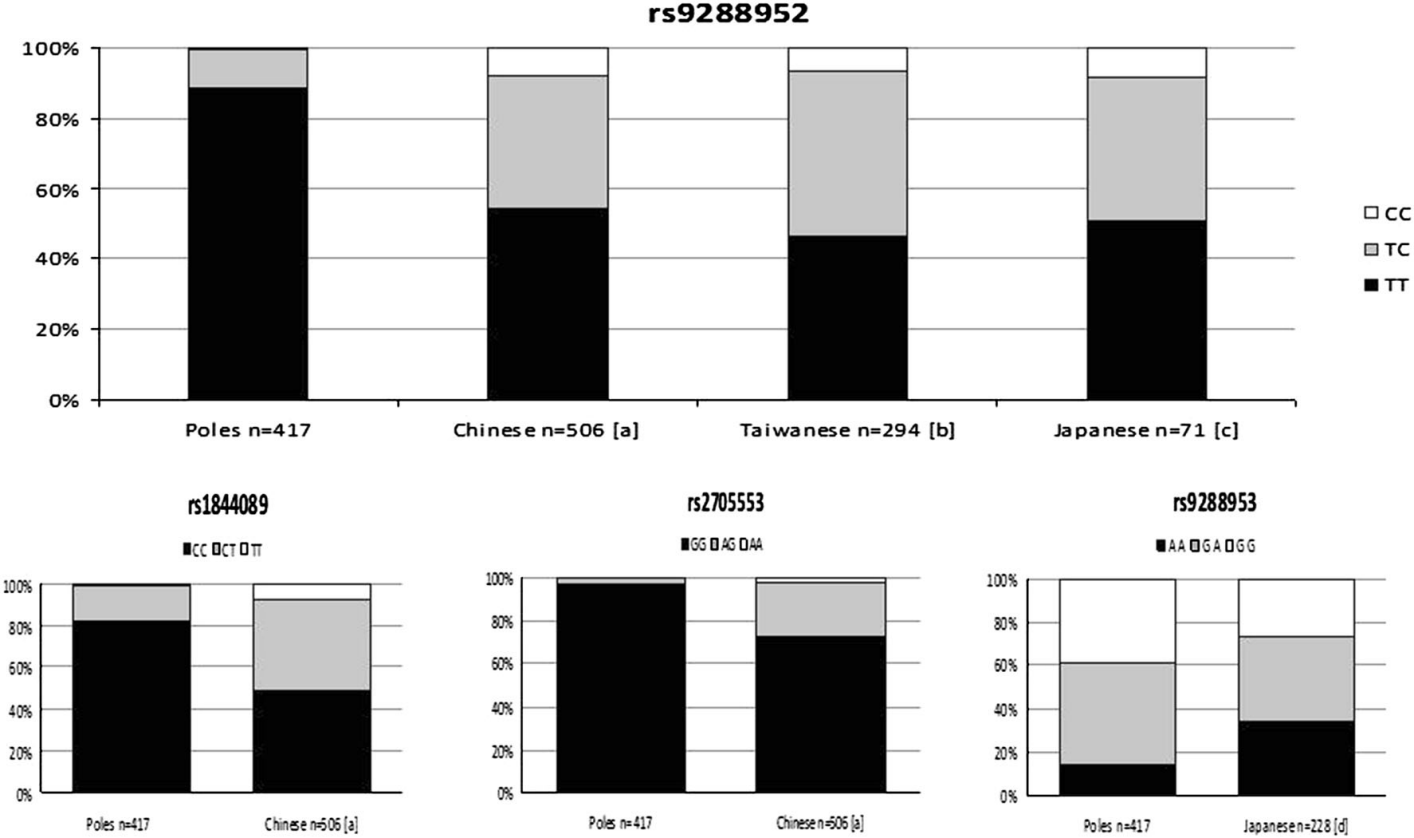
Distribution of genotypes for *BTLA* gene polymorphisms in different populations on the basis of literature survey. [a] Fu et al. (2010), [b] Lin et al. (2006), [c] Oki et al. (2011), [d] Inuo et al. (2009). Distributions of genotypes were significantly different between Polish population and others Asian population compared separately, and compared with Asian population together (*p* < 0.0000025; with Bonfrerroni corrections)

On the basis of a literature survey (Fu et al. 2010; Lin et al. 2006; Oki et al. 2011), we compared the genotype frequency for four SNPs (Fig. 2). The SNP rs9288952 was the most commonly investigated and there were data available from Chinese, Taiwanese and Japanese population. This polymorphism is situated in exon 4 and is a missense mutation. The nucleotide exchange causes amino acid residue change Pro-Leu in position 219. The literature data indicate that this SNP is associated with susceptibility to rheumatoid arthritis in the Japanese population and with breast cancer risk in Chinese women. We found statistically significant differences in genotype distribution between Polish and Chinese as well as Polish and Taiwanese populations (Fig. 2). For two other polymorphisms, rs1844089 and rs2705535, connected with breast cancer risk, only data from the Chinese population (Fu et al. 2010) were available. We found statistically significant differences in distribution of genotypes between Polish and Chinese populations.

The rs9288953 SNP was investigated in a Japanese population as a candidate SNP for type 1 diabetes mellitus or systemic lupus (Inuo et al. 2009). The study showed no associations of that polymorphism with selected autoimmune diseases. In our comparison, we found statistically different distribution of genotypes between Polish and Japanese healthy volunteers. Of note, the genotype distributions reported in this study for controls were not in HWE.

Recent papers have indicated the key role of the HVEM–BTLA pathway in inflammation, autoimmunity and infection immunity (del Rio et al. 2010; Shui et al. 2011) as well as immune tolerance, especially in aspects of allotransplantation (Hobo et al. 2012).

On the other hand, it was shown in an animal model that polymorphisms existing in the mouse *btla* gene were associated with differences in the Ig domain and BTLA expression pattern between different mouse strains and cell lines (Han et al. 2004; Hurchla et al. 2005). Therefore, polymorphisms existing in the human *BTLA* gene might influence the predisposition to immune-related disease. This work contributes to the overall knowledge of the presence and frequency of occurrence of allelic variants in the Caucasian population precisely in Polish population.

## Abbreviations

BTLA: B and T lymphocyte attenuator
CD: Cluster of differentiation
CTLA-4: Cytotoxic leukocyte antigen-4
HVEM: Herpes virus entry mediator
HWE: Hardy–Weinberg equilibrium
IL: Interleukin
ITIM: Immunoreceptor tyrosine-based inhibitory motif
NCBI: National Center for Biotechnology Information
PD-1: Programmed death-1
SHP: Src homology region 2 domain-containing phosphatase
SNP: Single-nucleotide polymorphism
Th: T helper cells
